# Virtual Touch Tissue Quantification of Acoustic Radiation Force Impulse: A New Ultrasound Elastic Imaging in the Diagnosis of Thyroid Nodules

**DOI:** 10.1371/journal.pone.0049094

**Published:** 2012-11-13

**Authors:** Yi-Feng Zhang, Hui-Xiong Xu, Yong He, Chang Liu, Le-Hang Guo, Lin-Na Liu, Jun-Mei Xu

**Affiliations:** 1 Department of Medical Ultrasound, Tenth People’s Hospital of Tongji University, Shanghai, People’s Republic of China; 2 Department of Medical Ultrasound, Yichang Central People’s Hospital, The First College of Clinical Medical Science, China Three Gorges University, Yichang, People’s Republic of China; Innsbruck Medical University, Austria

## Abstract

**Objective:**

Virtual touch tissue quantification (VTQ) of acoustic radiation force impulse (ARFI) is a new quantitative technique to measure tissue stiffness. The study was aimed to assess the usefulness of VTQ in the diagnosis of thyroid nodules.

**Methods:**

173 pathologically proven thyroid nodules in 142 patients were included and all were examined by conventional ultrasound (US), conventional elasticity imaging (EI) and VTQ of ARFI. The tissue stiffness for VTQ was expressed as shear wave velocity (SWV) (m/s). Receiver-operating characteristic curve (ROC) analyses were performed to assess the diagnostic performance. Intra- and inter-observer reproducibility of VTQ measurement was assessed.

**Results:**

The SWVs of benign and malignant thyroid nodules were 2.34±1.17 m/s (range: 0.61–9.00 m/s) and 4.82±2.53 m/s (range: 2.32–9.00 m/s) respectively (P<0.001). The mean SWV ratios between each nodule and the adjacent thyroid tissue were 1.19±0.67 (range: 0.31–6.87) for benign and 2.50±1.54 (range: 0.85–6.69) for malignant nodules (P<0.001). ROC analyses indicated that the area under the curve was 0.861 (95% CI : 0.804, 0.918) (P<0.001) for SWV and 0.831(95% CI : 0.761, 0.900)(P<0.001) for the SWV ratio. The cutoff points for the differential diagnosis were 2.87 m/s for SWV and 1.59 for SWV ratio. The sensitivity, specificity, accuracy, positive predictive value, and negative predictive value for EI were 65.9%, 66.7%, 66.5%, 40.3%, and 85.1%, respectively, and were 63.6%–75%, 82.2%–88.4%, 80.3%–82.1%, 58.9%–65.1%, and 87.7%–90.5%, respectively, for VTQ. The diagnostic value of VTQ is the highest for nodules >20 mm and lowest for those ≤10 mm. The correlation coefficients were 0.904 for intraobserver measurement and 0.864 for interobserver measurement.

**Conclusions:**

VTQ of ARFI provides quantitative and reproducible information about the tissue stiffness, which is useful for the differentiation between benign and malignant thyroid nodules. The diagnostic performance of VTQ is higher than that of conventional EI.

## Introduction

The incidence of thyroid nodules is about 33%–68% in general population and 5–15% of these nodules were malignant [1], [2]. In the recent 20 years, the prevalence of the thyroid carcinoma has increased in three folds [3]. Fine-needle aspiration (FNA) is the standard method to find out whether a thyroid nodule is malignant or not, and it has been shown to be the most cost-effective way to select patients for surgery with sensitivities of 54%–90% and specificities of 60%–96% for the detection of malignant lesions, whereas there are some suspicious cases in 5%–11% and some non diagnostic aspirates in 17%–22% nodules [4]. Recent studies focused on the evaluation of non-invasive ultrasound (US)-based methods with an aim to provide a supplement of FNA. High-frequency US has been proposed to be a complement to FNA for the differential diagnosis between benign and malignant thyroid nodules due to its characteristics such as noninvasiveness, easy-performance, no radiation, and ability to detect nonpalpable nodules. The US patterns such as hypoechoic nodule, spot microcalcifications, and the absence of halo sign are useful for predicting thyroid malignancy [5], [6], whereas conventional US becomes highly predictive of malignancy only when multiple patterns are simultaneously present in a thyroid nodule, and no US features have both a high sensitivity and a high positive predictive value (PPV) for thyroid cancer. The sensitivity of conventional US ranged from 69% to 75% and the PPV ranged from 41.8% to 94.2% [7]. Tissue stiffness is another feature that may reflect the nature of the thyroid nodule, with the malignant nodule tends to be hard and the benign nodule be soft [8], [9], [10]. Elasticity imaging (EI) of US, which provides qualitative or semi-quantitative reflection of the tissue stiffness, has emerged into clinical practice in recent years and gained promising results in improving the diagnostic performance of conventional US. However, conventional EI is apt to be influenced by several factors such as poor reproducibility and lack of quantitative information, thus its use is limited in clinic practice.

Acoustic radiation force impulse (ARFI) is a new modality that could evaluate the tissue stiffness quantitatively. When ARFI imaging was undergoing, tissue in the region of interest (ROI) is mechanically excited by using short-duration acoustic pulses to generate small (1–10 µm) localized tissue displacements [11]. The more elastic a tissue is, the more displacement it undergoes. The displacements result in shear-wave propagation away from the region of excitation and are tracked by using US correlation-based methods. By measuring the time to peak displacement at each lateral location, the shear wave velocity (SWV) within the tissue can be calculated (measured in m/s). Its quantitative implementation is named as virtual touch tissue quantification (VTQ), which gives an objective numerical evaluation of the tissue stiffness [12], [13]. ARFI has been performed in the tissue such as breast, liver, kidney and pancreas, and also been used to evaluate the characterization of atherosclerotic plaques, as well as monitor the results of radiofrequency ablation [14], [15], [16], [17], [18]. Recently, ARFI has been used to evaluate the thyroid nodules using a curved US probe at 4 MHz, whereas few studies using a high frequency linear probe has been reported [19]. As yet, no studies have compared the diagnostic value of ARFI and conventional EI and evaluated the reproducibility of ARFI in thyroid. This prospective study was aimed to assess the usefulness of VTQ of ARFI in the differential diagnosis between benign and malignant thyroid nodules, and the intra- and inter-observer reproducibility was also assessed.

## Patients and Methods

### Patients

**Figure 1:**
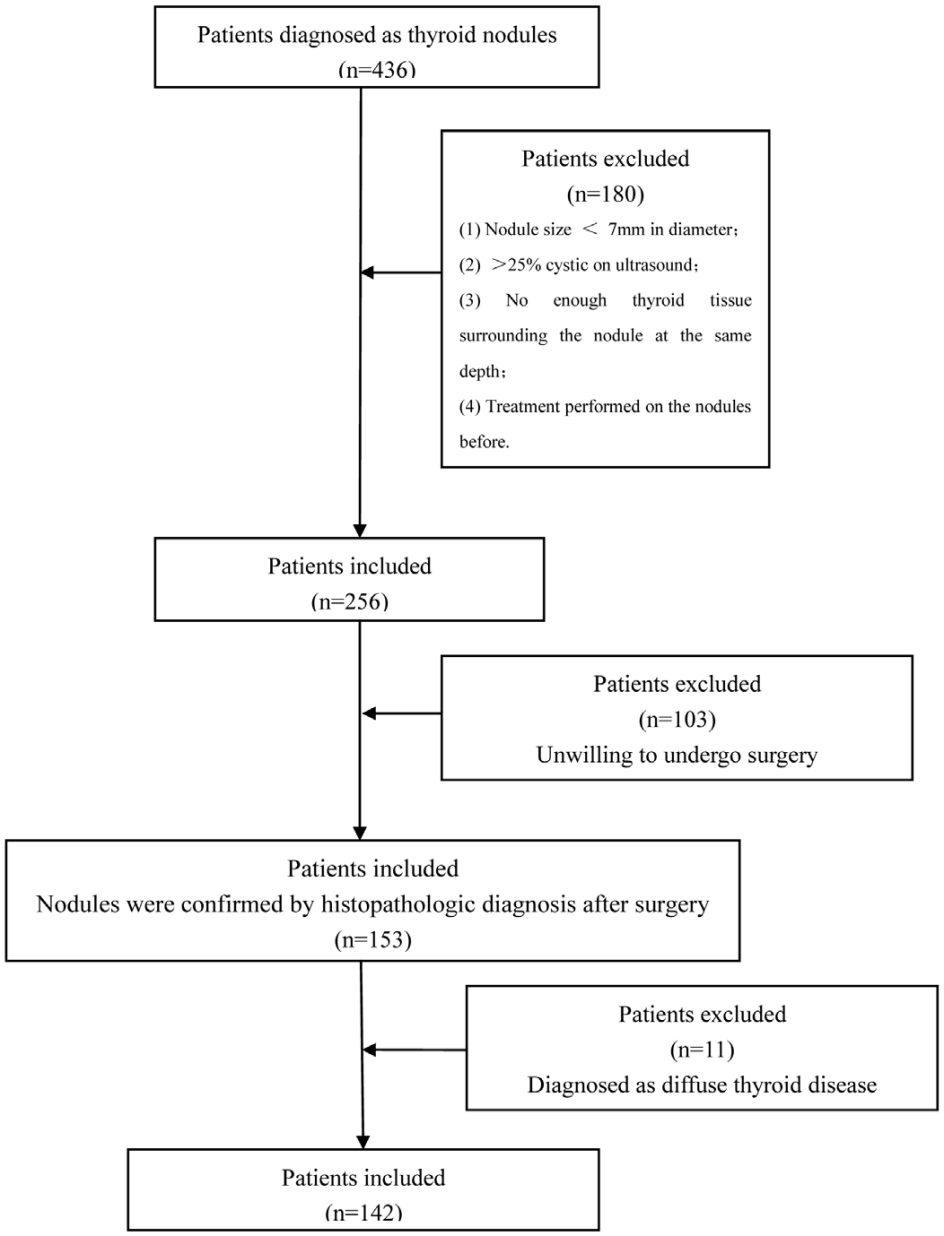
The flowchart of the selection of the patients with thyroid nodules.

From April 2011 to February 2012, 436 consecutive patients with thyroid nodules were detected on conventional US in the single center of a university hospital. Finally, 173 thyroid nodules in 142 patients with normal thyroid function were enrolled in the study, and all the nodules were confirmed by histopathology after surgery, including all the benign and malignant nodules. All the pathological diagnoses were made by one experienced pathologist. The flowchart for the patient selection was present in Figure 1. The enrollment criteria for the patients were as follows: (1) Nodule size larger than 7 mm in diameter, because the size of the sample ROI for ARFI is 6 mm×5 mm. (2) Solid or almost solid (<25% cystic) nodules on US [4]. (3) Enough thyroid tissue surrounding the nodule at the same depth. (4) No treatment performed on the nodules. For the patients who were unwilling to undergo surgery, FNA biopsy was carried out when the US findings were indeterminate or suspicious of malignancy. When the US findings were highly suggestive of benign nodules, such as spongiform on US, follow up with US examination every 6–12 months was carried out. The included patients were 42 men and 100 women. The patient age ranged from 16 to 75 yrs, and the mean age was 51±11 yrs. 33 patients had single nodule and 109 had multiple nodules in each. For the patients with multiple nodules, the nodules that were highly suspicious of malignancy or the largest one were selected for analysis. The diameter of the nodules ranged from 7 mm to 62 mm (mean, 19±11 mm).

The algorithm leading to surgery included those patients with compressive symptoms, nondiagnostic or indeterminate results on FNA, malignancy or suspicious malignancy on FNA. The types of the surgery were specified as follows. If the nodule was benign, different surgical approaches were carried out, including partial thyroid lobectomy, thyroid lobectomy, thyroid lobectomy with isthmusectomy and subtotal thyroidectomy, depending on the nodule number, size and location. If the nodule was found to be unilateral differentiated thyroid carcinoma, including occult thyroid carcinoma, thyroid lobectomy with isthmusectomy was taken. Thyroid carcinoma in the isthmus was resected by using the subtotal thyroidectomy, whereas total thyroidectomy was performed for bilateral differentiated thyroid carcinoma.

The clinical investigation has been conducted according to the principles expressed in the Declaration of Helsinki. According to the local legislation, oral informed consent was obtained from all patients older than 18 years old, whereas for the patients under the age of 18, oral informed consent was obtained from the next of kin, caretakers, or guardians on the behalf of the minors participants. The committee approved the consent procedures because the noninvasive technique used in this study was incorporated in a commercially available US machine and its safety has been well documented. The consent process was documented in a separate file after the oral informed consent was obtained. The study was approved by the Ethical Committee of the Tenth People’s Hospital of Tongji University.

### Conventional US

All studies were performed with the same S2000 US machine (Siemens Medical Solutions, Mountain View, CA, USA). A linear array transducer (9L4, Siemens Medical Solutions, Mountain View, CA, USA) with a center frequency of 7.5 MHz (range, 5.0–14.0 MHz) was equipped for all the B-mode US, EI, and ARFI elastography examinations. The ARFI technique was commercially available and was routinely used in clinical settings. All the nodules were examined by the same operator.

On conventional US, the following US features of the nodules were evaluated: echogenicity (hyper-, iso-, or hypoechogenicity with reference to adjacent thyroid parenchyma), presence or absence of halo sign, presence of spot microcalcifications (hyperechoic spots less than 2 mm, without acoustic shadowing). The color flow Doppler pattern was classified into three types: type I, absence of blood flow; type II, perinodular and absent or slight intranodular blood flow; type III, marked intranodular and absent or slight perinodular blood flow [5].

### Elasticity Imaging (EI)

Elasticity images were obtained after evaluating the lesions in conventional US using the same probe and by the same operator. The probe was placed on the neck gently, and a sampling box was highlighted by the operator, which included the nodule and sufficient surrounding thyroid tissue. A quality factor (QF) value greater than 60 over several frames is a key indicator of a good quality elastogram. Continuous scanning for approximately 10 seconds was performed and then the image was frozen. Afterwards, a US EI image, which was matched with an elasticity color scale, was obtained. The EI was classified using the Ueno and Ito [20] elasticity score: Score 1: Elasticity in the whole nodule. Score 2: Elasticity in a large part of the nodule. Score 3: Elasticity only at the peripheral part of the nodule. Score 4: No elasticity in the nodule. Score 5: No elasticity in the nodule and in the posterior shadowing. Score 4 and score 5 were regarded as malignant thyroid nodule, and score 1 to score 3 as benign thyroid nodule.

### ARFI Elastography

ARFI imaging was then performed on the long axis dimension of the nodule. ARFI imaging involves targeting an anatomic region to be interrogated for elastic properties with use of a ROI cursor while performing real-time B-mode imaging. Tissue in the ROI is mechanically excited by using short-duration acoustic pulses with a fixed transmission frequency to generate localized tissue displacement in tissue. The displacement results in shear-wave propagation away from the region of excitation and is tracked by using US correlation-based methods [21]. By measuring the time to peak displacement at each lateral location, the SWV is obtained. When performing ARFI, the probe was firstly placed gently on the body surface with light pressure on the thyroid. The patient was then asked to hold the breath and ARFI function was initiated. The basic principles for ROI selection are as follows: (1) ROI is placed on the solid portion of the nodule; (2) The calcified and liquefaction necrosis portions of the nodule are avoided; (3) The adjacent thyroid tissue is not included in ROI. The ARFI measurement was activated and the SWV value was displayed on the screen. After that, the ROI was moved to the surrounding thyroid tissue at the same depth and the procedure was repeated. The measurement was repeated for 7 times. The highest and the lowest value were eliminated and the mean of the rest 5 measurements was calculated and used for the analysis. The technique costs about 5 minutes for each nodule. It adds no additional cost for patient and it brings no inconvenience to the patients. The range for the SWV is 0–9 m/s. Value beyond these range is displayed as “x.xxm/s”, which means not applicable (NA). In other words, both extremely hard and soft tissue can be shown as“x.xxm/s”. After excluding the possible influencing factors such as patient’s respiration and operator’s inappropriate gesture, the value of “x.xxm/s” is allocated to be 0 m/s or 9 m/s with 0 m/s corresponding cystic portion and 9 m/s corresponding solid portion [15]. The SWV ratio between each nodule and adjacent thyroid tissue was also calculated. To investigate whether the nodule size would affect the diagnostic performance on thyroid nodules, the nodules were divided into three groups: Group 1: Nodule≤10 mm; Group 2: Nodule 11–20 mm; Group 3: Nodule>20 mm.

### Intra- and Inter-observer Reproducibility in ARFI Elastography

To investigate the intraobserver reproducibility, repeated SWV measurement for the same nodule was performed in 30 patients by the same operator within two days before surgery. Interobserver reproducibility was assessed independently by two operators, who made SWV measurement for the same nodule in 30 patients. Both of them had similar experience in use of the technique.

### Statistical Analysis

The statistical analyses were carried out using SPSS14.0 software package (SPSS Inc, Chicago, IL). P<0.05 were considered to be statistically significant. The differences between the mean SWV and SWV ratio of benign and malignant nodule were compared with independent t test. Analysis of variance was used to compare whether there were significant differences in the SWV and SWV ratio among different pathologic types. The intra- and inter-observer reproducibility was assessed using the correlation coefficient analysis. Qualitative data were compared with Chi-squared test. Receiver-operating characteristic curve (ROC) analyses were performed to assess the diagnostic performance of EI, as well as SWV and SWV ratio, in differentiating benign from malignant thyroid nodules. Areas under the receiver-operating characteristic curves (Az) were calculated and compared using the *z* test.

## Results

The basic characteristics of the patients and the US features of the nodules were presented in Table 1.

**Table 1:** The basic characteristics of the patients and the US features of the thyroid nodules.

	Benign	Malignant	*P*
Patient (n = 142)			
Sex (female/male)	70/34	30/8	0.178
Age (yrs)	51±12(16–75)	49±11 (27–72)	0.324
Single nodule/multiple nodule	20/81	13/28	0.128
Nodule (n = 173)			
Size (mm)	21±11 (7–62)	12±17 (7–40)	0.000
Location Left	58	25	0.096
Right	68	16	
Isthmus	3	3	
Echogenicity			0.000
Hyperechoic	6	0	
Iso-echoic	38	7	
Hypo-echoic	21	34	
Mixed solid and cystic	64	3	
Calcifications			0.000
None	98	19	
Microcalifications	14	23	
Macrocalcifications	17	2	

### Pathology

There were 129 benign nodules and 44 malignant nodules on pathology. Among the 129 benign nodules, 123 (95.3%) were nodular goiters and 6 (4.7%) were adenomas. The 44 malignant nodules were all papillary thyroid carcinomas.

### Conventional US

Absence of halo sign (sensitivity 90.9% and specificity 50.4%; *P*<0.001); hypoechoic nodule (sensitivity 98.0% and specificity 46.5%; *P*<0.001); spot microcalcifications (sensitivity 52.3% and specificity 89.1%; *P*<0.001) were the most predictive US patterns for malignancy. Intranodular blood flow was not predictive of malignancy (sensitivity 47.7% and specificity 56.6%; *P* = 0.619) (Table 2). Neither the combination of no halo sign and spot microcalcifications nor the combination of hypoechogenicity and spot microcalcifications increased the sensitivity or specificity compared with single conventional US feature. The absence of halo sign combined with the nodule hypoechogenicity was most predictive of malignancy (sensitivity 90.9% and specificity 82.2%; *P*<0.001).

**Table 2:** Predictive value of conventional US features in 173 thyroid lesions.

US features	BN(n)	CA(n)	Sensitivity(%)	Specificity(%)	PPV(%)	NPV(%)	Accuracy(%)	*P* value
Halo sign			90.9(40/44)	50.4(65/129)	38.4(40/104)	94.2(65/69	60.7(105/173)	0.000
Yes	65	4						
No	64	40						
Hypoechoic			98.0(43/44)	46.5(90/129)	52.4(43/82)	98.9(90/91)	76.9(133/173)	0.000
Yes	39	43						
No	90	1						
Spot microcalcifications			52.3 (23/44)	89.1(115/129)	62.2(23/37)	84.6(115/136)	79.8(138/173)	0.000
Yes	14	23						
No	115	21						
Type III vascularity			47.7 (21/44)	56.6 (73/129)	27.3 (21/77)	76.0 (73/96)	54.3 (94/173)	0.619
Yes	56	21						
No	73	23						
Combined features								
no halo sign +hypoechogenicity	23	40	90.9 (40/44)	82.2(106/129)	63.5(40/63)	96.4(106/110)	84.4(146/173)	0.000
no halo sign +spot microcalcification	4	22	50.0(22/44)	96.9(125/129)	84.6(22/26)	85.0(125/147)	85.0(147/173)	0.000
hypoechogenicity+ spot microcalcifications	4	22	50.0(22/44)	96.9(125/129)	84.6(22/26)	85.0(125/147)	85.0(147/173)	0.000

BN: benign; CA: carcinoma; PPV, positive predictive value; NPV, negative predictive value.

### Elasticity Imaging

EI score 1 was found in 16 nodules, no carcinoma; score 2 in 23 nodules, two carcinomas and 21 benign nodules; score 3 in 62 nodules, 13 carcinomas and 49 benign nodule; score 4 in 61 nodules, 26 carcinomas and 35 benign nodules; score 5 in 11 nodules, 3 carcinomas and 8 benign nodules. ROC curve analyses showed that the AUC for the EI was 0.663 (95% CI:0.569, 0.757) (*P* = 0.001). The sensitivity, specificity, accuracy, PPV and negative predictive value (NPV) in differentiating benign from malignant thyroid nodules were 65.9%, 66.7%, 66.5%, 40.3% and 85.1%, respectively.

### SWV and SWV Ratio of Thyroid Nodules

The mean SWVs of benign and malignant thyroid nodules were 2.34±1.17 m/s (range: 0.61–9.00 m/s) and 4.82±2.53 m/s (range: 2.32–9.00 m/s) respectively (*P*<0.001). For the benign nodules, the SWVs were ≤2.0 m/s in 49 (38.0%) nodules, 2.1–3.0 m/s in 62 (48.1%), and >3.0 m/s in 18 (13.9%), respectively. For the malignant nodules, the SWVs were ≤2.0 m/s in 0 (0.0%) nodules, 2.1–3.0 m/s in 14 (31.8%), and >3.0 m/s in 30 (68.2%), respectively. (*χ* ^2^ = 53.458, *P*<0.001).

The mean SWV ratios between each nodule and the adjacent thyroid tissue were 1.19±0.67 (range: 0.31–6.87) for the benign thyroid nodules and 2.50±1.54 (range: 0.85–6.69) for the malignant thyroid nodules respectively (*P*<0.001). For the benign nodules, the SWV ratios were ≤1.0 in 48 (37.2%) nodules, 1.1–2.0 in 76 (58.9%), and >2.0 in 5 (3.9%), respectively. For the malignant nodules, the SWV ratios were ≤1.0 in 2 (4.5%) nodules, 1.1–2.0 in 23 (52.3%), and >2.0 in 19 (43.2%), respectively (*χ* ^2^ = 48.903, *P*<0.001).

**Figure 2:**
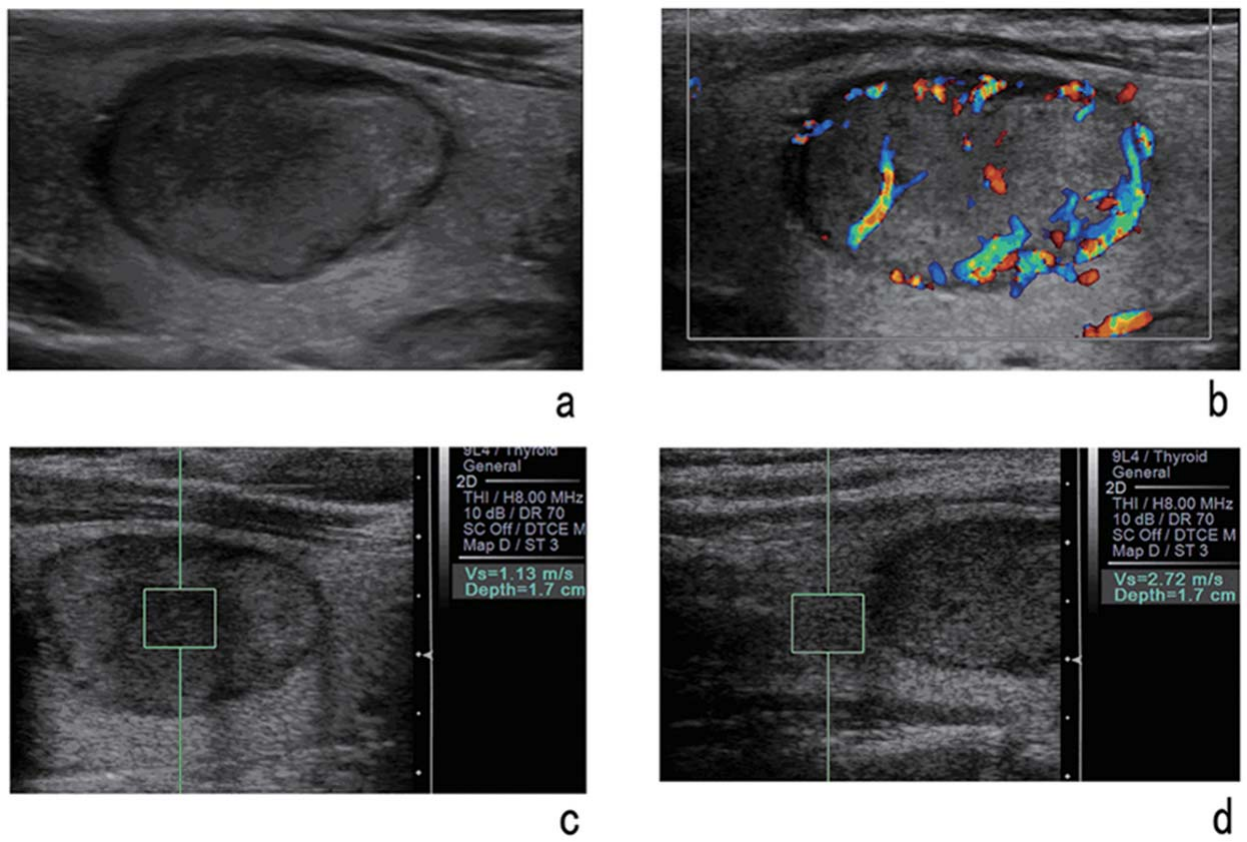
A 42-year-old woman with a solid lesion of nodular goiter. Gray-scale image (a) and color Doppler image (b) of the nodule. VTQ of the nodule (c) shows the SWV is 1.13 m/s and the SWV of the adjacent thyroid tissue (d) is 2.72 m/s.

**Figure 3:**
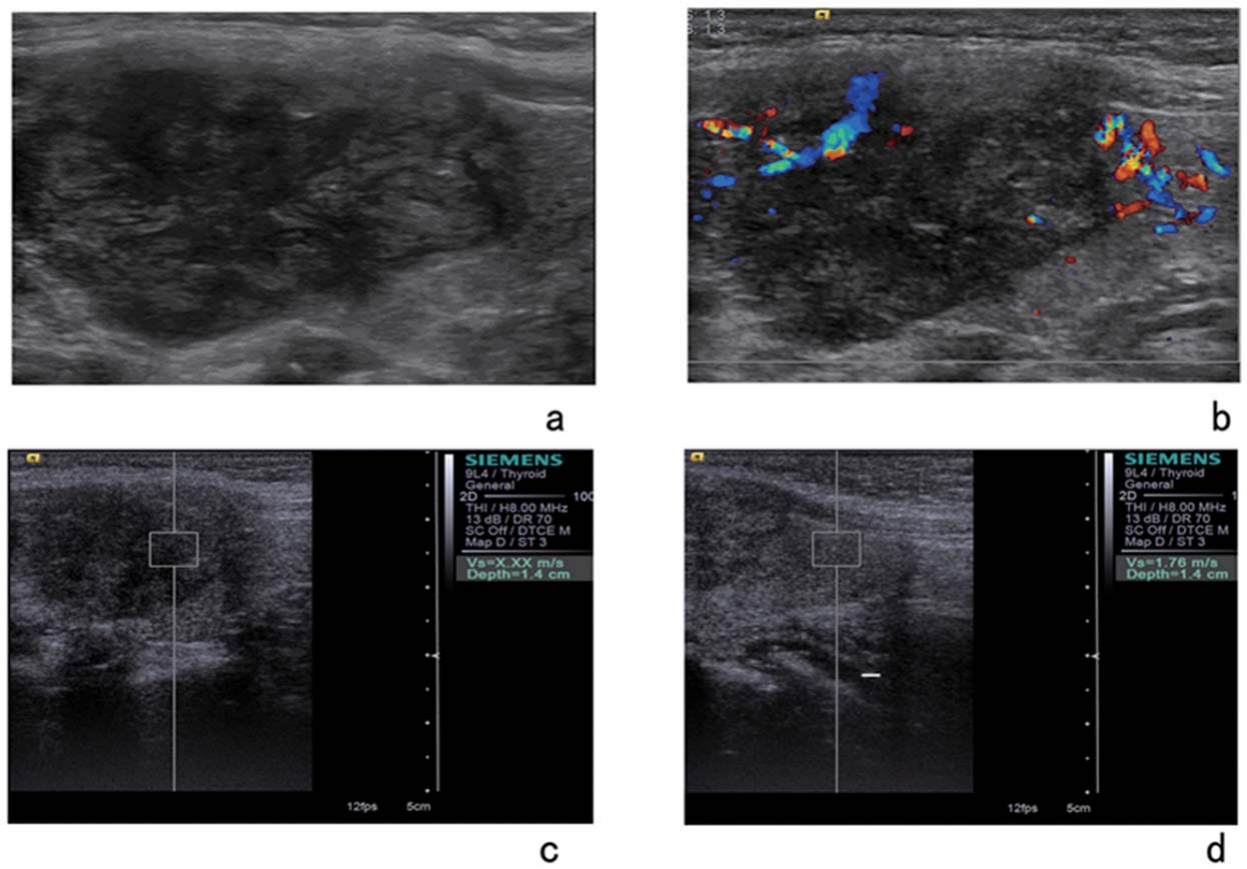
A 59-year-old woman with a solid lesion of papillary carcinoma. Gray-scale image (a) and color Doppler image (b) of the nodule. VTQ of the nodule shows the SWV is displayed as x.xxm/s and the SWV of the adjacent thyroid tissue (d) is 1.76 m/s.

With regard to different pathologic types, **t**he mean SWVs and SWV ratios were 2.46±1.75 m/s (range: 0.61–9.00 m/s) and 1.22±0.78 (range: 0.31–6.87) for nodular goiter, 2.25 m/s±1.53 m/s (range: 1.72 –3.78 m/s) and 1.15±0.67 (range: 0.84–2.66) for adenoma, and 4.82±2.53 m/s (range: 2.32–9.00 m/s) and 2.50±1.54 (range: 0.85–6.69)) for papillary thyroid carcinoma, respectively (Figure 2, 3). There were significant differences in SWV between papillary thyroid carcinoma and any other benign nodule in SWV (compared with nodular goiter, *P*<0.001; adenoma, *P*<0.001) and SWV ratio (compared with nodular goiter, *P*<0.001; adenoma, P = 0.002), but there were no significant differences between the groups of benign nodules (P>0.05).

ROC curve analyses showed that the AUCs for the SWV were 0.861 (95% CI : 0.804, 0, 918; *P*<0.001) (Z = 3.07, *P*<0.01, in comparison with EI) and 0.831(95% CI : 0.761, 0.900; *P*<0.001) (Z = 2.50, *P*<0.05, in comparison with EI) for the SWV ratio in differentiating benign from malignant thyroid nodules. The best cutoff points were 2.87 m/s for SWV and 1.59 for SWV ratio. The sensitivity, specificity, accuracy, PPV, NPV and accuracy for SWV were present in Table 3.

**Table 3:** The diagnostic performance of SWV and SWV ratio in differentiating benign from malignant thyroid nodules.

Nodules	Method	Sensitivity%	Specificity%	PPV%	NPV%	Accuracy%	AUC
≤10 mm	SWV	68.2(15/22)	74.9(17/23)	71.4(15/21)	70.8(17/24)	71.1(32/45)	0.707
	SWV ratio	81.8(18/22)	52.2(12/23)	62.1(18/29)	75(12/16)	66.7(30/45)	0.672
11–20 mm	SWV	94.1(16/17)	70.7(29/41)	57.1(16/28)	96.7(29/30)	77.6(45/58)	0.879
	SWV ratio	76.5(13/17)	87.8(36/41)	72.2(13/18)	90(36/40)	84.5(49/58)	0.862
>20 mm	SWV	100(5/5)	84.6(55/65)	33.3(5/15)	100(55/55)	85.7(60/70)	0.940*
	SWV ratio	80(4/5)	98.5(64/65)	80(4/5)	98.5(64/65)	97.1(68/70)	0.914**
Total	SWV	75(33/44)	82.2(106/129)	58.9(33/56)	90.5(106/117)	80.3(139/173)	0.861
	SWV ratio	63.6(28/44)	88.4(114/129)	65.1(28/43)	87.7(114/130)	82.1(142/173)	0.831

* Compared with AUC of SWV in nodule ≤10 mm, *z* = 2.73, *P<*0.01.

** Compared with AUC of SWV ratio in nodule ≤10 mm, *z* = 2.30, *P<*0.05.

When dividing the nodules into three groups with different size, for the nodules ≤10 mm, AUC was 0.707 with SWV (*P* = 0.018, 95% CI : 0.553, 0.860) and 0.672 (*P* = 0.048, 95% CI : 0.514, 0.830) with the SWV ratio; for the nodules sized 11–20 mm, 0.879 with SWV (*P*<0.001, 95% CI : 0.791, 0.966) and 0.862 with SWV ratio(*P* = 0.000, 95% CI : 0.751, 0.972); for the nodules >20 mm, 0.940 with SWV (*P* = 0.001, 95% CI : 0.858, 1.000) and 0.914 (*P* = 0.002, 95% CI : 0.000, 1.000) with SWV ratio. There were significant differences between nodules ≤10 mm and those >20 mm with regard to AUC for the SWV (Z = 2.73, *P<*0.01), as well as for SWV ratio (Z = 2.30, *P<*0.05), whereas no significant differences were found between any other two groups (all *P*>0.05). The corresponding sensitivity, specificity, accuracy, PPV, and NPV were presented in Table 3.

### Reproducibility in ARFI Elastography

The intraobserver difference in measuring SWV was 0.28±0.20 m/s (range: 0.02 to 0.97 m/s) and the interobserver difference was 0.32±0.26 m/s (range: 0.02 to 1.00 m/s). The correlation coefficients were 0.904 for intraobserver measurement and 0.864 for interobserver measurement (both *P<*0.001).

## Discussion

FNA is the most accurate and cost effective method for evaluating thyroid nodules. The main limitation of FNA cytology of thyroid nodules is the substantial proportion of indeterminate lesions and nondiagnostic aspirates. Among patients with a cytological diagnosis of indeterminate lesion, 25% display a final diagnosis of malignancy on histology [22]. The recommended treatment of indeterminate nodules remains the surgical excision of the nodule. As for nondiagnostic cytology, the surgical treatment maybe indicated based on clinical or US features suggestive of malignancy, or on compressive symptoms due to the nodular size [8]. The sensitivity and specificity of US in differentiating malignant from benign thyroid nodules are various, highly depending on the skillfulness of the operators [23], [24]. The American Association of Clinical Endocrinologists and Associazione Medici Endocrinologi guidelines recommend US-guided FNA for nodules >10 mm, US-FNA is suggested for nodules <10 mm only if clinical information or US features are suspicious [25]. Therefore, a reliable, noninvasive method to tell a thyroid nodule is malignant or not is demanded, beyond the conventional US. EI of US has been proposed to evaluate the thyroid nodules and the sensitivity, specificity, PPV and NPV for the diagnosis of malignant thyroid nodules were reported to be 86–97%, 85–100%, 95–100% and 98–100%, respectively [26], [27]. However, in a recent study, Lippolis et al [28] found that the PPV and NPV of EI were 34% and 50%. The low performance was clinically negligible and it was suggested the need for quantitative analytical assessment of nodule stiffness to improve the efficacy of EI. The uncertainty for EI is largely due to that EI is a qualitative and operator-depending procedure. ARFI is a new technique of elastography that has been recently introduced into clinical practice and can overcome the limitations of previous elastography techniques [29], [30], [31], [32]. In the present study, we firstly compared the value of EI and ARFI in the differential diagnosis between benign and malignant thyroid nodules. The diagnostic value of EI was found to be relatively lower. Friedrich-Rusthad et al [19] carried out a feasibility study to evaluate ARFI in the thyroid gland using a curved US probe at 4 MHz. Their studies included 49 benign nodules and 3 malignant ones and the specificity of ARFI imaging for the diagnosis of malignant thyroid nodules was 91–95%.

In the present study, the SWV value of malignant nodules was significantly higher than that of benign nodules. VTQ showed a relatively high and balanced sensitivity and specificity (75% and 82.2%) for identifying malignancy in thyroid nodules. The sensitivity, specificity, positive predictive value, negative predictive value, and diagnostic accordance rate of VTQ were reported to be 86.36%, 93.42%, 79.17%, 95.95% and 91.84% respectively in the study of Gu et al [33].The best SWV cutoff point was 2.87 m/s in the present study, similar with 2.55–3.30 m/s reported in the previous studies [19], [33]. These findings suggested that it may be useful to introduce ARFI elastography into routine clinical practice. The SWV ratio was firstly introduced in the present study, which took into account the influence of the background of thyroid tissue. After adding the SWV ratio for analysis, the specificity increased from 71% to 88% for nodules 11––20 mm and from 85% to 99% for nodules >20 ; in addition, the PPV increased from 57.1% to 77.2% for nodules 11–20 mm and from 33.3% to 80% for nodules >20 mm, as compared with SWV. These results indicated the additional usefulness of SWV ratio.

The diagnostic value of VTQ was associated with the nodule size, with relatively high value for nodules >20 mm in diameter and inferior value for those≤10 mm in diameter. The pathological components in nodules ≤10 mm might be different from those >20 mm, which in turn leads to different stiffness. Therefore, further study was mandatory to evaluate the relationship between SWV and the pathological components in the thyroid nodule. The diagnostic inadequacy of VTQ in the nodules ≤10 mm also indicated that VTQ should be used in combination with conventional US features instead of VTQ alone.

In the present study, although the comparison between conventional US and VTQ of ARFI was not performed. The 173 nodules were evaluated by two independent readers with consensus using a five-point scale of confidence level, that is definitely benign, probably benign, indeterminate, probably malignant, and definitely malignant. By using the conventional US, the numbers of lesions with an allocated confidence level of definitely benign, probably benign, indeterminate, probably malignant, and definitely malignant were 67 (38.7%), 13 (7.5%), 46(26.6%), 46 (26.6%), and 1 (0.6%), respectively. After adding VTQ of ARFI for analysis, the corresponding values were 81 (46.8%), 31 (17.9%), 19 (11.0%), 25 (14.5%), and 17 (9.8%), respectively (not shown in the results). The indeterminate lesions decreased significantly after review of VTQ images, which indicated that VTQ would improve the confidence levels of the operators.

The present study has some limitations. In this series, the solitary nodules outweighed the number of the patients with multinodular goiter that there were 33 patients with solitary nodules and 109 patients with multiple nodules, because only the solid or almost solid nodules were included in this prospective study. Hence the results are only suitable for such a clinical scenario. Secondly, the influence of chronic thyroiditis to stiffness is an issue of concern, whereas it was excluded in this study. However, Magri et al have found that the elasticity of thyroid nodules was independent from the coexistence of autoimmune thyroiditis [34]. Thirdly, although the upper limit of “x.xxm/s”was mainly found in malignant thyroid nodules, it was found in two benign nodules. The pathology results of the two benign nodular goiters showed that interstitial fibrous tissue, collagen and calcifications were present, which also indicated that SWV may be related to the pathological components. Finally, this study was only a preliminary exploratory study, thus there was no evidence that this technique added to the value of FNA and whether VTQ could be included to be part of the decision-tree for surgery. Future prospective multi-center studies with large case series are mandatory to evaluate the additional usefulness of this technique to FNA and the role of this technique in the decision-tree for the treatment of thyroid nodules.

There are some technical concerns related to VTQ measurement in thyroid. Firstly, the thyroid gland is closed to the carotid artery and the pulsation of carotid artery might affect the measurement. Secondly, if the stiffness of the tissue beyond the limits of measurement, whether high or low, the SWV would be displayed as “x.xxm/s”, which made the calculation of mean SWV difficult. Finally, the VTQ can only reveal the local stiffness of the nodule, which might cause sample errors in practice. Therefore, VTQ should be applied in combination with other techniques including conventional US.

In conclusion, VTQ of ARFI imaging provides quantitative and reproducible information about the tissue stiffness, which is useful for differentiating between benign and malignant thyroid nodules. The diagnostic performance of VTQ is higher than those of conventional EI. On the other hand, the diagnostic value of VTQ of ARFI in thyroid nodules ≤1.0 cm is not satisfactory. Larger prospective studies are needed to confirm the role of this new technique in the decision-tree in selecting patients for surgery.

## References

[1] Cooper DS, Doherty GM, Haugen BR, Kloos RT, Lee SL, et al (2006) Management guidelines for patients with thyroid nodules and differentiated thyroid cancer. Thyroid 16: 109–142. 16420177 10.1089/thy.2006.16.109

[2] Sebag F, Vaillant-Lombard J, Berbis J, Griset V, Henry JF, et al (2010) Shear wave elastography: A new ultrasound imaging mode for the differential diagnosis of benign and malignant thyroid nodules. J Clin Endocrinol Metab 95: 5281–5288. 20881263 10.1210/jc.2010-0766

[3] Remontet L, Estève J, Bouvier AM, Grosclaude P, Launoy G, et al (2003) Cancer incidence and mortality in France over the period 1978–2000. Rev Epidemiol Sante Publique 51(1 Pt 1): 3–30. 12684578

[4] Berker D, Aydin Y, Ustun I (2008) The value of fine-needle aspiration biopsy in subcentimeter thyroid nodules. Thyroid 18: 603–608. 18578608 10.1089/thy.2007.0313

[5] Rago T, Vitti P, Chiovato L, Mazzeo S, De Liperi A, et al (1998) Role of conventional ultrasonography and color flow-Doppler sonography in predicting malignancy in “cold” thyroid nodules. Eur J Endocrinol 138: 41–46. 9461314 10.1530/eje.0.1380041

[6] Papini E, Guglielmi R, Bianchini A, Crescenzi A, Taccogna S, et al (2002) Risk of malignancy in nonpalpable thyroid nodules: predictive value of ultrasound and color-Doppler features. J Clin Endocrinol Metab 87: 1941–1946. 11994321 10.1210/jcem.87.5.8504

[7] Frates MC, Benson CB, Charboneau JW, Cibas ES, Clark OH, et al (2005) Management of thyroid nodules detected at US: Society of Radiologists in Ultrasound consensus conference statement. Radiology 237: 794–800. 16304103 10.1148/radiol.2373050220

[8] Cooper DS, Doherty GM, Haugen BR, Kloos RT, Lee SL, et al (2006) American Thyroid Association Guidelines Taskforce 2006 Management guidelines for patients with thyroid nodules and differentiated thyroid cancer. Thyroid 2: 2–33. 10.1089/thy.2006.16.109 16420177

[9] Pacini F, Schlumberger M, Dralle H, Elisei R, Smit JW, et al (2006) European Thyroid Cancer Taskforce 2006 European consensus for the management of patients with differentiated thyroid carcinoma of the follicular epithelium. Eur J Endocrinol 154: 787–803. 16728537 10.1530/eje.1.02158

[10] Gharib H, Papini E, Valcavi R, Baskin HJ, Crescenzi A, et al (2006) American Association of Clinical Endocrinologists and Associazione Medici Endocrinologi medical guidelines for clinical practice for the diagnosis and management of thyroid nodules. Endocr Pract 12: 63–102. 10.4158/EP.12.1.63 16596732

[11] Nightingale K, Soo MS, Nightingale R, Trahey G (2002) Acoustic radiation force impulse imaging: in vivo demonstration of clinical feasibility. Ultrasound Med Biol 28: 227–235. 11937286 10.1016/s0301-5629(01)00499-9

[12] D’Onofrio M, Gallotti A, Pozzi Mucelli R (2010) Virtual Touch tissue quantification: measurement repeatability and normal values in the healthy liver. AJR Am J Roentgenol 195: 132–136. 20566806 10.2214/AJR.09.3923

[13] Gallotti A, D’Onofrio M, Pozzi Mucelli R (2010) Acoustic radiation force impulse (ARFI) technique in ultrasound with virtual touch tissue quantification of the upper abdomen. Radiol Med 115: 889–897. 20082227 10.1007/s11547-010-0504-5

[14] Kwon HJ, Kang MJ, Cho JH, Oh JY, Nam KJ, et al (2011) Acoustic radiation force impulse elastography for hepatocellular carcinoma-associated radiofrequency ablation. World J Gastroenterol 17: 1874–1878. 21528062 10.3748/wjg.v17.i14.1874 PMC3080723

[15] Meng W, Zhang G, Wu C, Wu G, Song Y, et al (2011) Preliminary results of acoustic radiation force impulse (ARFI) ultrasound imaging of breast lesions. Ultrasound Med Biol 37: 1436–1443. 21767903 10.1016/j.ultrasmedbio.2011.05.022

[16] Yu H, Wilson SR (2011) Differentiation of benign from malignant liver masses with acoustic radiation force impulse technique. Ultrasound Q 27: 217–223. 22124386 10.1097/RUQ.0b013e318239422e

[17] Stock KF, Klein BS, Vo Cong MT, Sarkar O, Römisch M, et al (2010) ARFI-based tissue elasticity quantification in comparison to histology for the diagnosis of renal transplant fibrosis. Clin Hemorheol Microcirc 46: 139–148. 21135489 10.3233/CH-2010-1340

[18] Yashima Y, Sasahira N, Isayama H, Kogure H, Ikeda H, et al (2012) Acoustic radiation force impulse elastography for noninvasive assessment of chronic pancreatitis. J Gastroenterol 47: 427–432. 22065162 10.1007/s00535-011-0491-x

[19] Friedrich-Rust M, Romenski O, Meyer G, Dauth N, Holzer K, et al (2012) Acoustic radiation force impulse-imaging for the evaluation of the thyroid gland: a limited patient feasibility study. Ultrasonics 52: 69–74. 21788057 10.1016/j.ultras.2011.06.012

[20] Ueno E, Ito A (2004) Diagnosis of breast cancer by elasticity imaging. Eizo Joho Medical 36: 2–6.

[21] Nightingale K, Soo MS, Nightingale R, Trahey G (2002) Acoustic radiation force impulse imaging: in vivo demonstration of clinical feasibility. Ultrasound Med Biol 28: 227–235. 11937286 10.1016/s0301-5629(01)00499-9

[22] Rago T, Di Coscio G, Basolo F, Scutari M, Elisei R, et al (2007) Combined clinical, thyroid ultrasound and cytological features help to predict thyroid malignancy in follicular and Hupsilonrthle cell thyroid lesions: results from a series of 505 consecutive patients. Clin Endocrinol (Oxf) 66: 13–20. 17201796 10.1111/j.1365-2265.2006.02677.x

[23] Lagalla R, Caruso G, Novara V, Cardinale AE (1993) Flowmetric analysis of thyroid diseases: hypothesis on integration with qualitative color-Doppler study. Radiol Med 85: 606–610. 8327762

[24] Papini E, Guglielmi R, Bianchini A, Crescenzi A, Taccogna S, et al (2002) Risk of malignancy in nonpalpable thyroid nodules: predictive value of ultrasound and color-Doppler features. J Clin Endocrinol Metab 87: 1941–1946. 11994321 10.1210/jcem.87.5.8504

[25] Gharib H, Papini E, Paschke R, Duick DS, Valcavi R, et al.. (2010) American Association of Clinical Endocrinologists, Associazione Medici Endocrinologi, and European Thyroid Association Medical Guidelines for Clinical Practice for the Diagnosis and Management of Thyroid Nodules. Endocr Pract 16(Suppl 1): 1–43. 10.4158/10024.GL 20497938

[26] Gietka-Czernel M, Kochman M, Bujalska K (2010) Real-time ultrasound elastography - a new tool for diagnosing thyroid nodules. Endokrynol Pol 61: 652–657. 21104638

[27] Bojunga J, Herrmann E, Meyer G (2010) Real-time elastography for the differentiation of benign and malignant thyroid nodules: a meta-analysis. Thyroid 20: 1145–1150. 20860422 10.1089/thy.2010.0079

[28] Lippolis PV, Tognini S, Materazzi G, Polini A, Mancini R, et al (2011) Is elastography actually useful in the presurgical selection of thyroid nodules with indeterminate cytology? J Clin Endocrinol Metab 96: E1826–E1830. 21865373 10.1210/jc.2011-1021

[29] Lyshchik A, Higashi T, Asato R (2005) Thyroid gland tumor diagnosis at US elastography. Radiology 237: 202–211. 16118150 10.1148/radiol.2363041248

[30] Garra BS, Cespedes EI, Ophir J (1997) Elastography of breast lesions: initial clinical results. Radiology 202: 79–86. 8988195 10.1148/radiology.202.1.8988195

[31] Cochlin DL, Ganatra RH, Griffiths DF (2002) Elastography in the detection of prostatic cancer. Clin Radiol 57: 1014–1020. 12409113 10.1053/crad.2002.0989

[32] Rago T, Santini F, Scutari M, Pinchera A, Vitti P (2007) Elastography: new developments in ultrasound for predicting malignancy in thyroid nodules. J Clin Endocrinol Metab 92: 2917–2922. 17535993 10.1210/jc.2007-0641

[33] Gu J, Du L, Bai M, Chen H, Jia X, et al (2012) Preliminary study on the diagnostic value of acoustic radiation force impulse technology for differentiating between benign and malignant thyroid nodules. J Ultrasound Med 31: 763–771. 22535724 10.7863/jum.2012.31.5.763

[34] Magri F, Chytiris S, Capelli V, Alessi S, Nalon E, et al (2012) Shear wave elastography in the diagnosis of thyroid nodules: feasibility in the case of coexistent chronic autoimmune Hashimoto’s thyroiditis. Clin Endocrinol (Oxf) 76: 137–41. 21740455 10.1111/j.1365-2265.2011.04170.x

